# Laser-Assisted Preparation of TiO_2_/Carbon/Ag Nanocomposite for Degradation of Organic Pollutants

**DOI:** 10.3390/ma17164118

**Published:** 2024-08-20

**Authors:** Shahin Almasi Nezhad, Babak Jaleh, Elham Darabi, Davoud Dorranian

**Affiliations:** 1 Plasma Physics Research Center, Science and Research Branch, Islamic Azad University, Tehran 1477893855, Iran; shahinalmasi626@gmail.com (S.A.N.); edarabi@yahoo.com (E.D.); doran@srbiau.ac.ir (D.D.); 2 Department of Physics, Faculty of Science, Bu-Ali Sina University, Hamedan 6517838695, Iran

**Keywords:** lignin-derived carbon, TiO_2_ nanoparticles, Ag particles, laser ablation, organic dyes

## Abstract

The ever-increasing expansion of chemical industries produces a variety of common pollutants, including colors, which become a global and environmental problem. Using a nanocatalyst is one of the effective ways to reduce these organic contaminants. With this in mind, a straightforward and effective method for the production of a novel nanocatalyst based on lignin-derived carbon, titanium dioxide nanoparticles, and Ag particles (TiO_2_/C/Ag) is described. The preparation of carbon and Ag particles (in sub-micro and nano size) was carried out by laser ablation in air. The nanocomposite was synthesized using a facile magnetic stirrer of TiO_2_, C, and Ag. According to characterization methods, a carbon nanostructure was successfully synthesized through the laser irradiation of lignin. According to scanning electron microscope images, spherical Ag particles were agglomerated over the nanocomposite. The catalytic activities of the TiO_2_/C/Ag nanocomposite were tested for the decolorization of methylene blue (MB) and Congo red (CR), employing NaBH_4_ in a water-based solution at 25 °C. After adding fresh NaBH_4_ to the mixture of nanocomposite and dyes, both UV absorption peaks of MB and CR completely disappeared after 10 s and 4 min, respectively. The catalytic activity of the TiO_2_/C/Ag nanocomposite was also examined for the reduction of 4-nitrophenol (4-NP) using a NaBH_4_ reducing agent, suggesting the complete reduction of 4-NP to 4-aminophenol (4-AP) after 2.30 min. This shows excellent catalytic behavior of the prepared nanocomposite in the reduction of organic pollutants.

## 1. Introduction

Growing population and human activity have provided important environmental issues. Water pollution, which can create fundamental problems for living organisms, has become one of the most discussed problems in the world. Consuming water sources in industrial, domestic, and agricultural sections has led to water pollution consisting of different combinations, such as dyes, heavy metals, and pesticides [1]. The released dye effluents from the textile, paper and pulp section, leather tanning, and dye production industries provide significant dangerous impacts on the environment and human well-being [2]. Dyes are colored molecules consisting of chromophores and auxochromes and have complex structures [3]. Dyes can cause respiratory and skin disorders, as well as contact dermatitis [1]. Since Congo red (CR) is commonly utilized across various sections, including textiles, paper production, and printing, it can be significantly released into aquatic systems as pollution. It is also highly toxic and carcinogenic. This anionic dye cannot easily degrade through common methods because of its benzene and naphthalene rings. It has significant optical, thermal, and physicochemical stability because of its aromatic structure. Therefore, its removal needs effective methods [4]. Methylene blue (MB) is the predominant dye found in the textile and pharmaceutical sections, recognized widely as a cationic dye. Although it does not have high toxic effects, it can provide significant issues, such as breathing difficulties, puking, runs, and qualms [1,5]. Hence, the development of environmentally friendly, easy, applicable, and high-efficiency methods for the elimination of dye residues from industrial effluents and other water sources is necessary to mitigate the adverse impacts of dyes on the environment and organisms. In brief, several chemical, physical, and biological methods have been used for dye removal from water sources, in which electrochemical treatment, adsorption, oxidation, and membrane filtration stand as the crucial techniques for achieving this objective [3,6]. Physical methods are common methods because of their simple set up and efficiency, in which the least amount of chemicals is needed compared with chemical and biological methods. In chemical methods, chemistry theories are utilized for dye removal. Biological methods are affordable and facile to operate as an alternative for dye removal, in which bacteria, fungi, yeast, and algae are used for the bacterial degradation of dyes. However, these methods are not enough to completely remove hazardous particles from dye wastewater. Therefore, colored water can still be seen. These methods are eco-friendly, produce a lower amount of sludge, and can treat the chemical oxygen demand (COD) in wastewater. The main disadvantage of biological methods is their growth rate, because they deal with living things. In comparison with physical and biological methods, chemical methods are not common in industries due to their costly setup and operation. Another disadvantage of chemical methods is the production of toxic pollutions during the dye removal process, creating additional disposal problems [2,7]. The removal percentage range was reported as 88.8–99, 76–90.1 and 86.8–99% for chemical, biological and physical methods, respectively [2]. One of the most practical methods is catalytic dye reduction through the reducing agent of NaBH_4_, which can effortlessly be carried out in medium conditions using suitable catalysts [8]. Nitroarene derivatives are widely employed to produce textile dyes, pharmaceutical drugs, and pesticides. Among them, 4-nitrophenol (4-NP) is a highly hazardous organic contaminant for human health and the environment. This can release into the environment as wastewater of dyeing and pharmaceutical industries. The reduction of 4-NP to a less toxic amine derivative of 4-aminophenol (4-AP) using nanocatalysts is a well-known approach for converting pollution into a high-value product [9,10].

Aiming to further reduce 4-NP and remove/degrade toxic dyes, using metal nanoparticles such as platinum, gold, palladium, silver, and copper has garnered recent attention due to their remarkable physical and chemical properties, as well as their efficacy in color removal [11,12,13,14]. Ag NPs have been shown to have high catalytic activity and optical properties. The main advantage of using Ag NPs compared with other metallic NPs is their cost-effectiveness and high stability under ambient conditions. They can also be synthesized through various chemical, physical, and biological methods. Although chemical methods can produce Ag NPs at a high yield, these methods need detrimental chemicals, while physical methods do not need to use any chemicals [15,16]. Laser ablation is a rapid, affordable, controllable, and eco-friendly physical method for preparing Ag NPs. The shape and size of the synthesized nanoparticles can be controlled by adjusting laser parameters, including wavelength, power, pulse number, and repetition rate. It can be carried out in water and air environments. Unlike the air environment, the laser ablation of the Ag target in water produces a mixture of metallic Ag NPs and Ag oxide NPs [9]. Accordingly, laser ablation in the air is employed as a physical method for preparing Ag NPs in this research. Recently, loading metallic NPs on inorganic supports such as TiO_2_ NPs, graphene oxide, and Fe_3_O_4_ has been developed to prepare heterogeneous catalytic systems [17,18]. Among these, TiO_2_ NPs, stable metal oxides with significant properties, such as nontoxicity, hydrophilicity, and low cost, are appropriate for removing hazardous compounds from water [19,20]. TiO_2_ consists of three crystallin phases of anatase, rutile, and brookite. Among these phases, anatase has attracted much attention due to its tendency to absorb different organic compounds [21].

Lignin constitutes a significant portion of the cell walls in natural lignocellulosic plants and is recognized as the second most abundant natural polymer on Earth, following cellulose. It has a complex structure that is relevant to its source. In addition, it can be produced through pulp and paper industries with a high yield. In the industrial process, lignin has been burnt to produce power. In addition, lignin has received widely attention for producing high-value materials due to its biocompatibility, negligible toxicity, and environmental friendliness [22,23]. Lignin consists of a high ratio of carbon to oxygen and can be employed as an ideal carbon precursor [22,24,25]. Since lignin is a soft polymer, it can be used to prepare carbon with an adjusted porous structure and morphologies. This process produces high-value products and generates income for the pulping industry and biorefineries [26,27]. According to reports, template methods have been employed to fabricate porous carbon derived from lignin [27,28]. In this research, a simple and single-step method, the laser ablation of lignin, was utilized for generating carbon in ambient conditions. It was reported that the composition of TiO_2_ NPs with carbon-based materials has significant applications, such as the detection of vapors [29], biomedical applications [30], photocatalyst preparation [31,32], and catalysts [33,34]. The preparation of nanocatalysts, including TiO_2_ NPs, Ag NPs, and carbon-based materials, has provided significant performance toward dye removal. For example, Nasrollahzadeh et al. reported that Ag/RGO/TiO_2_ nanocomposites show higher catalytic activity for MB (immediately), CR (116 s), and 4-NP (3.15 min) removal in aqueous media at an ambient temperature than that of GO/TiO_2_ [35]. In fact, the composition of TiO_2_ NPs with carbon materials can create support with a high surface area and high adsorption ability of dyes and other pollutants, leading to more reactant molecules near to Ag NPs.

In this work, simple and eco-friendly methods were employed to fabricate nanocomposite, including TiO_2_ NPs, lignin-derived carbon, and Ag particles. Laser ablation in air was used as a fast and straightforward method for preparing Ag particles and lignin-derived carbon. To prepare the nanocomposite, TiO_2_ NPs, Ag particles, and C were exclusively sonicated and then mixed with a magnetic stirrer to obtain a uniform suspension. The characterization of the nanocomposite represents that the amorphous carbon and metallic Ag particles can be successfully generated using a laser ablation process at ambient conditions. The nanocomposite’s catalytic activity was assessed in the removal process of MB and CR, with the presence of a NaBH_4_ reduction agent. The catalytic reduction of 4-NP was examined in the presence of TiO_2_/C/Ag catalyst. 

## 2. Materials and Methods

### 2.1. Materials and Equipment

To prepare carbon, lignin was prepared from Iran. To generate Ag nanoparticles, a high-purity silver plate (99.9%) served as the target for the laser ablation process. TiO_2_ nanoparticles containing biphasic anatase and rutile phases in an 80 to 20 ratio were procured from Degussa Co. (Essen, North Rhine-Westphalia, Germany). A fiber laser (RFLP30Q, Raycus, Wuhan, China, 1064 nm, 100 ns, 30 W) was employed for the laser ablation process. The crystalline structure of samples was examined via X-ray diffraction (XRD, Philips, PW 1730, λ = 1.54 Å). Fourier transform infrared (FT-IR, Thermo Nicolet 370 pellet spectrometer, USA) spectra of samples were detected to evaluate the chemical bonds and functional groups. To identify detailed information about the chemical structure of samples, the Raman spectrum was measured with Raman device (Teksan, Takram P50C0R10, Tehran, Iran), with laser beam wavelength of 532 nm. X-ray photoelectron spectroscopy (XPS, SPECS, Berlin, Germany) was utilized to identify chemical bonding state and elements on the nanocomposite surface. The morphology and dispersion of the particles were examined using a field emission scanning electron microscope (FESEM, TESCAN-MIRA3-XMU, Brno, Czech Republic) and a scanning electron microscope (SEM, JEOL-JSM-840A, Tokyo, Japan). The surface area and pore diameter of the nanocomposite were analyzed using Brunauer–Emmett–Teller (BET) and Barret–Joyner–Halenda (BJH) techniques, respectively, with the BELSORP-mini II instrument (Japan). UV-Vis spectral analysis (Hitachi, Tokyo, Japan, U-2900) was used to assess the catalytic degradation of organic dyes.

### 2.2. Preparation of Carbon Powder

**Figure 1:**
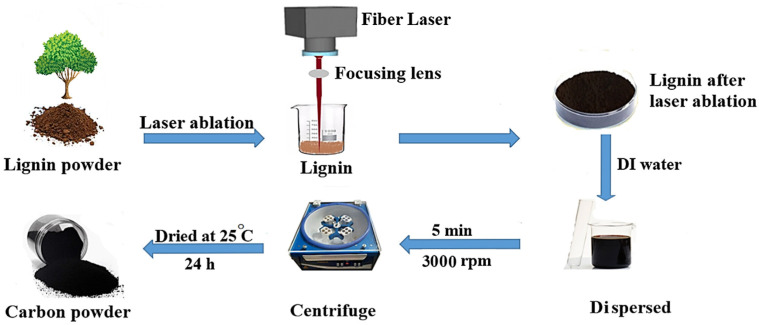
Schematic of the preparation of carbon powder using laser ablation of lignin.

To fabricate the carbon structure, laser ablation in air was used. First, 0.1 g of lignin powder was poured into a beaker with a volume of 5 mL and covered with an appropriate glass container. Then, it was subjected to fiber laser irradiation with a scanning speed of 400 mm/s and 15 W laser output power. In order to prevent excessive temperature increase and maintain the uniformity of the laser effect on the target material, this process was stopped every 1 min, the powder was stirred with the help of a spatula, and laser irradiation was repeated, resulting in a black powder. To remove the retained lignin or impurities in the final product, the black powder was dispersed in an appropriate volume of deionized (DI) water and then centrifuged at 3000 rpm for 5 min. The colloidal carbon obtained was subsequently dried at room temperature. A schematic illustration of the carbon preparation process is depicted in Figure 1.

### 2.3. Preparation of TiO_2_/Carbon/Ag Nanocomposite

The laser ablation in the air process was used to prepare Ag particles. Firstly, the Ag plate was placed in a closed glass container. The laser beam (27 W and scanning speed of 200 mm/s) was focused on an area of 9 mm × 9 mm on the silver plate. After 30 min irradiation, the gray Ag particles powder was formed on the container wall and could be collected.

**Figure 2:**
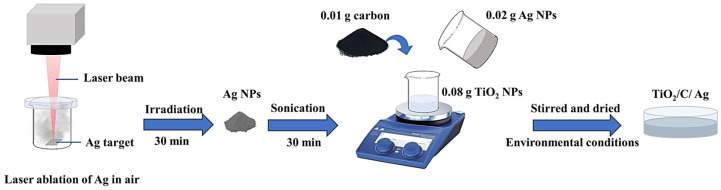
Schematic of TiO_2_/C/Ag nanocomposite preparation process.

To prepare the nanocomposite, 0.02 g of Ag particles was uniformly dispersed in 10 mL of deionized (DI) water through sonication for 30 min. In addition, 0.08 g of TiO_2_ NPs was dispersed in 20 mL of DI water for 30 min under sonication. The dispersed Ag particles were then added to TiO_2_ NPs suspension. After that, 0.01 g of the prepared carbon was added to the TiO_2_/Ag suspension, then stirred for one day to obtain a uniform suspension, and finally dried at ambient conditions. The prepared nanocomposites were introduced as TiO_2_/C/Ag. Figure 2 depicts a schematic of the nanocomposite preparation process.

### 2.4. Investigation of the Dye Removal Efficiency of TiO_2_/C/Ag Nanocomposite

#### 2.4.1. The Catalytic Reduction of Methylene Blue (MB)

The reduction of MB was conducted under ambient conditions. A total of 0.008 g of the TiO_2_/C/Ag nanocomposite was added to a 25 mL solution of MB (3.1 × 10^−5^ M). Subsequently, 25 mL of fresh NaBH_4_ aqueous solution (5.3 × 10^−3^ M) was rapidly introduced into the mixture at 25 °C. The progress of the catalytic reduction of MB was tracked using UV-Vis spectroscopy at a wavelength of 664 nm. Reduction of MB in the presence of TiO_2_/C and without catalyst (in the presence of NaBH_4_) was also performed.

#### 2.4.2. The Catalytic Reduction of Congo Red (CR)

For the catalytic reduction of CR using the TiO_2_/C/Ag nanocomposite, 25 mL of NaBH_4_ solution (5.3 × 10^−^^3^ M) was added to an aqueous medium containing 0.008 g of TiO_2_/C/Ag and 25 mL of CR solution (1.44 × 10^−^^5^ M) at room temperature. The catalytic reduction of CR was assessed through UV-Vis spectroscopy, where a decrease in absorbance intensity at λ_max_ = 498 nm indicated the reduction of CR. To investigate catalyst recyclability, the nanocomposite was washed with H_2_O several times and then used for another cycle. Reduction of CR was also tested without catalyst and in the presence of TiO_2_/C.

#### 2.4.3. The Catalytic Reduction of 4-NP

A total of 0.008 g of the TiO_2_/C/Ag nanocomposite was added to aqueous solution of 4-NP (2.5 × 10^−^^3^ M, 25 mL). Then, freshly produced NaBH_4_ (250 mM, 25 mL) was added into the mixture. The reduction of 4-NP was observed through changing solution’s yellow color and studied using UV-Vis spectroscopy at wavelengths of 300 and 400 nm. It is worth mentioning that 4-NP reduction was also investigated without catalyst and in the presence of TiO_2_/C.

## 3. Results

### 3.1. Characterization

**Figure 3:**
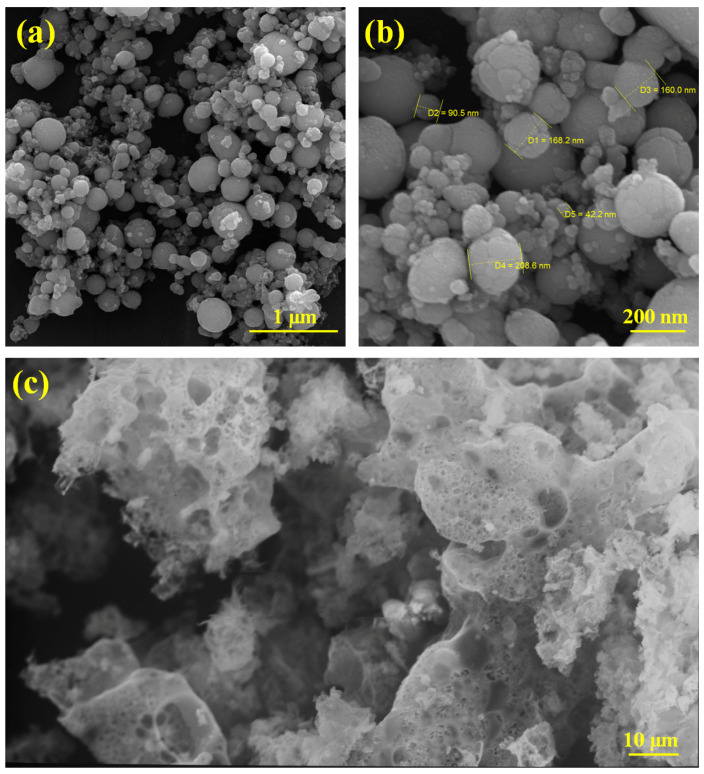
(**a**,**b**) FESEM images of Ag particles and (**c**) SEM image of carbon.

A morphological investigation of Ag particles and carbon was performed using FESEM and SEM images, as illustrated in Figure 3. The prepared Ag particles are almost spherical (Figure 3a,b). According to Figure 3b, Ag particles in nano and sub-micro size can be obtained using the laser ablation process. Figure 3c indicates the SEM image of the prepared carbon, which confirms the carbon’s porous structure.

**Figure 4:**
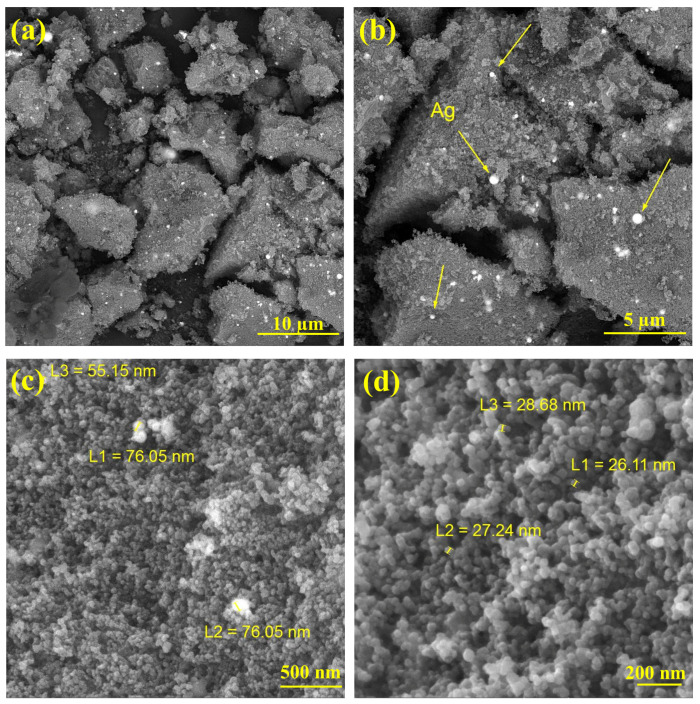
FESEM micrographs of the TiO_2_/C/Ag nanocomposite on different scales of (**a**) 10 µm, (**b**) 5 µm, (**c**) 500 nm and (**d**) 200 nm.

**Figure 5:**
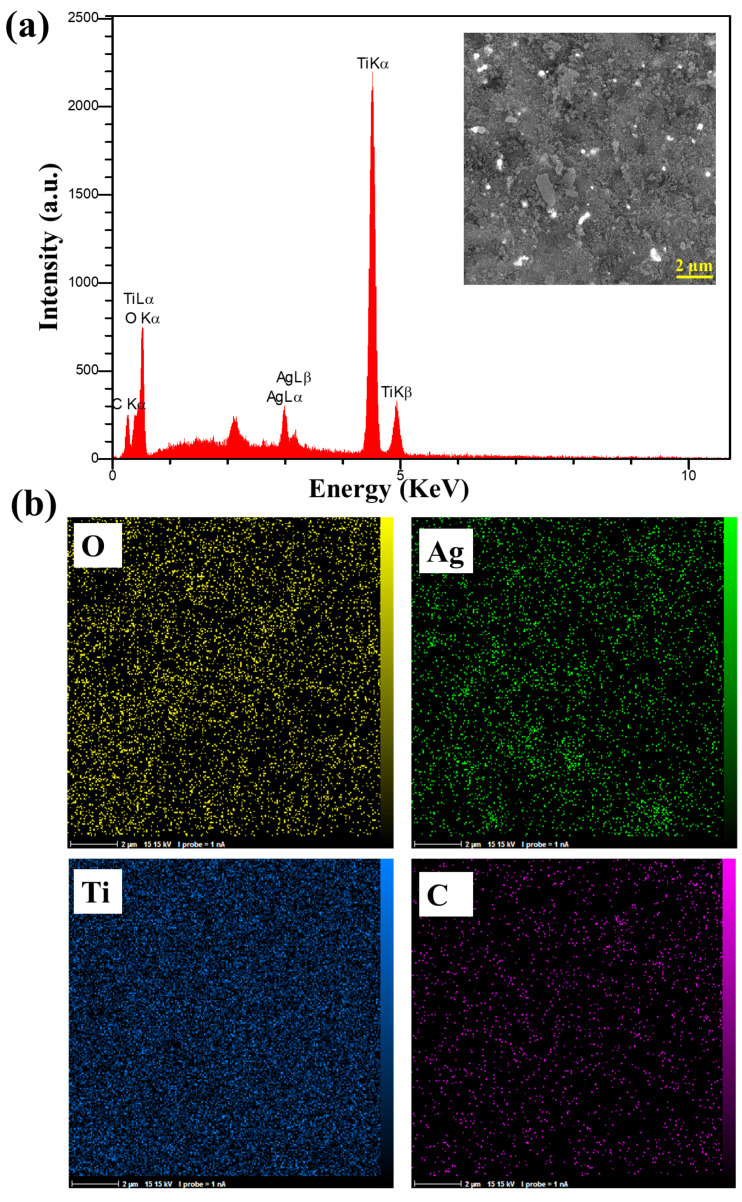
(**a**) EDS spectrum and (**b**) elemental mapping images of the TiO_2_/C/Ag nanocomposite.

The FESEM analysis shown in Figure 4 examines the morphology of the nanocomposite. A rough and agglomerated structure can be observed in Figure 4a. As shown in Figure 4b, the white spots indicate the precipitated Ag particles, which are spherical and agglomerated in some places. Figure 4d shows TiO_2_ NPs with a diameter of about 30 nm, which have been connected to each other. Based on the EDS spectrum (Figure 5a), the emission peaks of Ag, C, O, and Ti elements only indicate the presence of carbon, TiO_2_, and Ag in the sample. The elemental mapping analysis (Figure 5b) shows the elemental distribution, where the Ag particles are almost regularly distributed in the sample.

**Figure 6:**
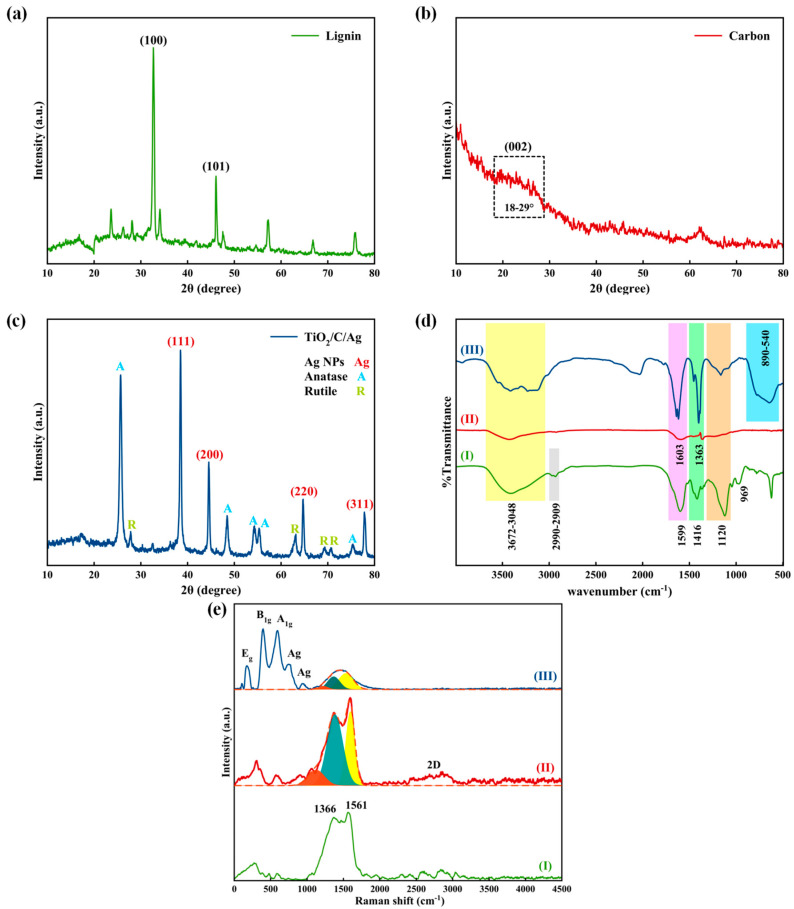
XRD patterns of (**a**) lignin, (**b**) carbon, and (**c**) TiO_2_/C/Ag nanocomposite; (**d**) FT−IR and (**e**) Raman spectra of (I) lignin, (II) carbon, and (III) TiO_2_/C/Ag nanocomposite.

The crystallographic structure of lignin, carbon, and the prepared nanocomposite was evaluated through XRD patterns, as depicted in Figure 6a–c. The lignin XRD pattern shows two sharp peaks at 32.67° and 45.97° due to diffraction from (100) and (101) planes, indicating the crystal nature of lignin [36,37,38]. According to Figure 6b, the laser ablation process creates amorphous carbon, which can be confirmed with a shoulder appearing at the 2θ range of 18–29°, related to the (002) plane [39]. The XRD pattern of the as-prepared TiO_2_/C/Ag nanocomposite is shown in Figure 6c. Four sharp peaks at 38.46°, 44.49°, 64.64°, and 77.80° are characteristic of Ag particles, which are attributed to the diffraction of (111), (200), (220), and (311) planes (JCPDS card no. 04-0783), respectively. The presence of the anatase phase of TiO_2_ NPs (JCPDS card no. 04-0477) can be confirmed through diffraction peaks at 25.61°, 48.46°, 54.19°, 55.28°, and 75.36°, corresponding to the planes of (101), (200), (105), (211), and (215), respectively, while weak peaks located at 27.80°, 69.33°, 63.18°, and 70.66° show rutile phase of TiO_2_ (JCPDS card no. 21-1276), corresponding to (110), (301), (002), and (112), respectively. The high-intensity peak observed at 25.61° indicates that anatase is the primary phase present in the TiO_2_ NPs.

To identify chemical bonds and functional groups, FT-IR analysis was carried out at a wavenumber of 500–4000 cm^−1^. A broad absorption peak appeared at 3672–3048 cm^−1^, indicating -OH stretching [40]. Absorption peaks at the wavenumber range of 2990–2909 cm^−1^ and 969 cm^−1^ exhibit the presence of C-H bonding vibration. A peak located at 1120 cm^−1^ is characteristic of C-O stretching vibration [36]. Four absorption peaks at 1364 cm^−1^, 1416 cm^−1^, 1599 cm^−1^, and 1603 cm^−1^ are attributed to the C=C vibration bond [41,42]. The presence of TiO_2_ NPs indicates a broad absorption peak at the wavenumber range of 890-524 cm^−1^ attributed to Ti-O vibration [43,44]. According to Figure 6d(III), no absorption peak related to Ag particles is observed, indicating non-chemical interaction between nanostructures.

As shown in Figure 6e, Raman spectroscopy was performed to provide detailed information about the chemical structure of lignin, lignin-derived carbon, and the TiO_2_/C/Ag nanocomposite. The peaks located at 1366 cm^−1^ and 1561 cm^−1^ are due to the C-H and aromatic ring stretching of lignin [45]. As shown in Figure 6e(II), a broad peak located in the range of 960–1761 cm^−1^ can be fitted with three peaks exhibiting T, D, and G bands. The peak located at 1380 cm^−1^ is attributed to the D band, suggesting double-resonance related to a chaotic carbonaceous structure (or A_1g_ vibration mode that is characteristics of sp^3^ defects). The G band (1592 cm^−1^) is due to the in-plane vibration of E_2g_ in sp^2^-bonded graphitic carbon [46,47]. A T band is also present at 1140 cm^−1^, which is related to impurities or heteroatoms [48,49]. After the laser ablation of lignin, a 2D peak with low intensity appeared at about 2720 cm^−1^, showing graphitic domains in the lignin-derived carbon [50]. According to the fitted peaks, the content of the D band (54.82%) was higher than the G band (32.55%), suggesting a disordered structure with defects for the prepared carbon [49,51]. As shown in the TiO_2_/C/Ag Raman spectrum, two peaks at 170 cm^−1^ and 397 cm^−1^ are attributed to E_g_ and B_1g_, respectively, indicating Raman’s active mode of the anatase phase, while the rutile phase of TiO_2_ NPs is determined through a peak at 597 cm^−1^ related to A_1g_ [52,53]. Two peaks located at 751 cm^−1^ and 942 cm^−1^ are assigned to Ag particles [54].

**Figure 7:**
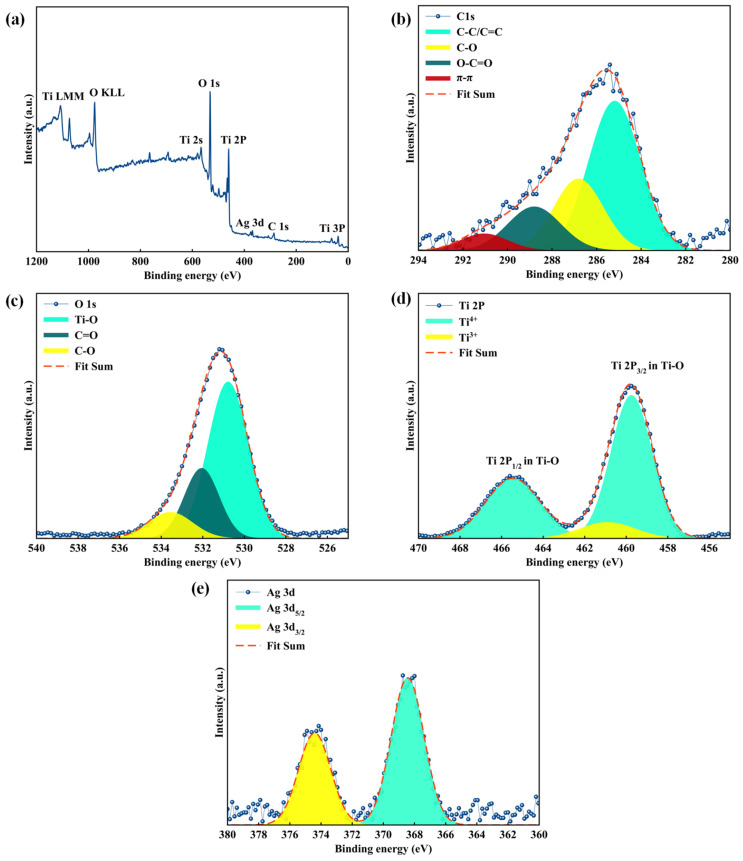
(**a**) XPS survey spectra and high-resolution XPS spectra of (**b**) C1s, (**c**) O1s, (**d**) Ti2p, and (**e**) Ag3d of TiO_2_/C/Ag nanocomposite.

XPS analysis was employed to determine the chemical states of the elements and compositions of the sample (Figure 7). Figure 7a indicates the survey spectra of TiO_2_/C/Ag nanocomposite that confirm the presence of C, O, Ti, and Ag elements. The high-resolution spectrum of C1s can be deconvoluted into four peaks at binding energies of 285.2, 286.8, 288.8, and 291.0 eV, consisting of C-C/C=C, C-O, O-C=O, and π-π binding, respectively (Figure 7b) [55,56,57]. According to Figure 7c, the deconvolution of O1s suggests the presence of lattice oxygen of Ti-O (530.7 eV), C=O (532.1 eV), and C-O (533.5 eV) bonding [58,59]. As shown in Figure 7d, two sharp peaks located at 459.7 and 465.4 eV indicate Ti2p_3/2_ and Ti2p_1/2_ of Ti^4+^, respectively, suggesting the presence of the anatase phase, while a shoulder at 460.8 eV is due to the presence of Ti^3+^ species [60,61,62,63]. According to the Ag3d spectrum, two strong peaks at 374.4 and 368.4 eV attributed to Ag3d_3/2_ and Ag3d_5/2_ can be observed, indicating Ag(0) [64,65].

**Figure 8:**
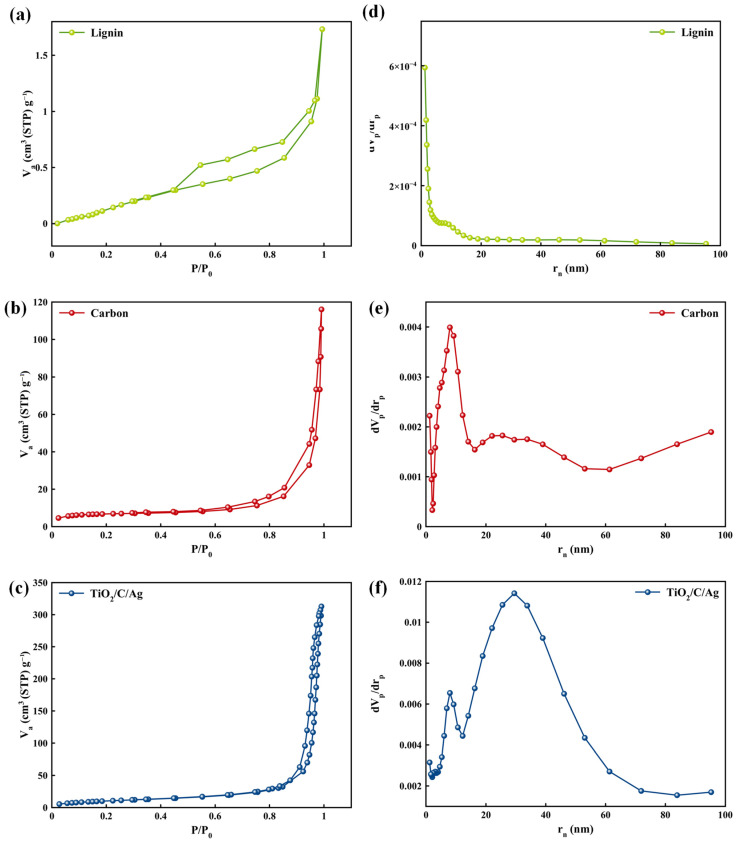
(**a**–**c**) N_2_ adsorption/desorption at 77 k and (**d**–**f**) pore structure of samples.

To evaluate the surface area and pore size distribution of samples, BET and BJH analyses were performed (Figure 8). As depicted in Figure 8a–c, the N_2_ adsorption/desorption isotherm of the samples can be presented as type IV, indicating a type H3 hysteresis loop. This confirms a mesoporous structure for all samples [66,67,68,69]. The maximum pore diameters of 1.2, 7.9, and 29.5 nm were achieved for lignin, lignin-derived carbon, and the TiO_2_/C/Ag nanocomposite, respectively. Information about the surface structure of the samples is further detailed in Table 1. Using lignin as a carbon source can be effective for preparing porous carbon structures with significant surface areas. It is noteworthy to mention that the composition of lignin-derived carbon with TiO_2_ NPs and Ag particles can increase surface properties, especially surface area. It can provide active sites for dye removal.

**Figure 9:**
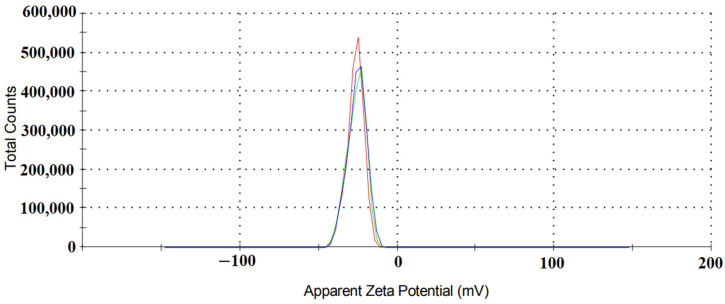
Zeta potential analysis of TiO_2_/C/Ag nanocomposite.

The zeta potential of the TiO_2_/C/Ag nanocomposite was measured to identify the surface charge of the catalyst, respectively. As shown in Figure 9, the zeta potential of the TiO_2_/C/Ag was obtained as −25.5 mV, suggesting a high dispersity and good colloidal nature of the catalyst. It indicates that the catalyst surface is negatively charged [70,71].

The optical band gap of TiO_2_ and the TiO_2_/C/Ag nanocomposite can be calculated through the Tauc equation as follows [72,73]:

(1) $${\left(\alpha h\nu \right)}^{r}=E_{D}(h\nu -E_{g})$$

**Figure 10:**
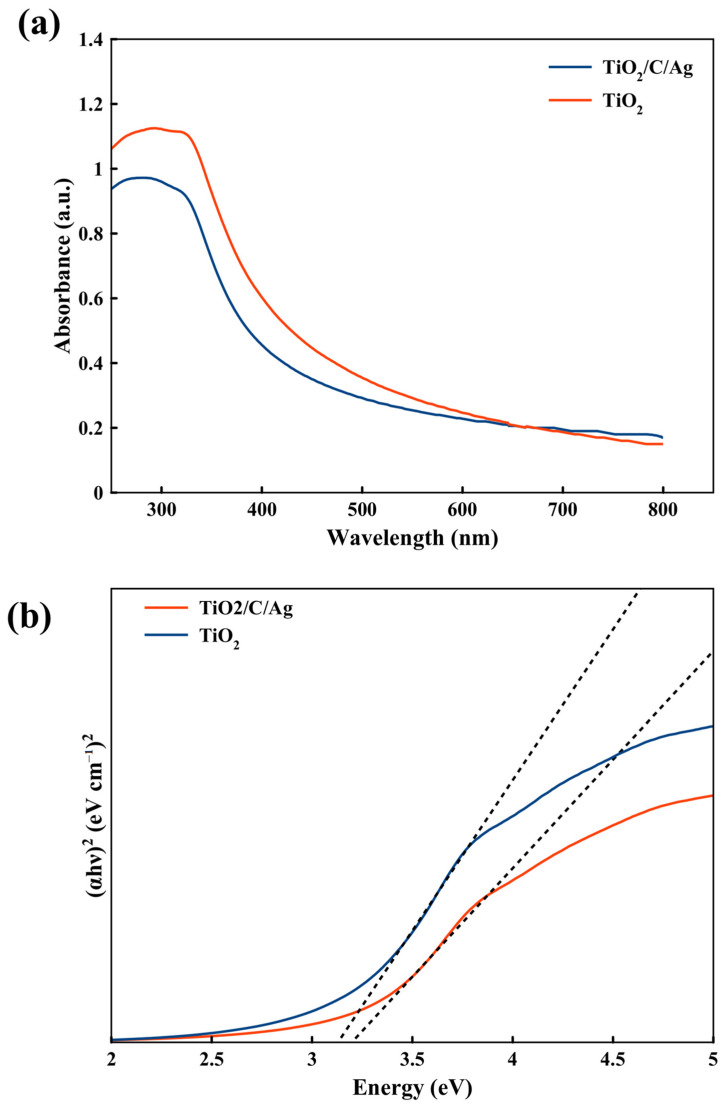
(**a**) UV-Vis spectra of TiO_2_ and TiO_2_/C/Ag nanocomposite, and (**b**) the Touc plot for TiO_2_ and TiO_2_/C/Ag nanocomposite.

Here, *α*, *hν*, *E_g_*, and *E_D_* are the optical absorption coefficient, photon energy, optical band gap, and a constant, respectively. r indicates the nature of the electron transition and can be 2 or 1/2 for the direct or indirect transition band openings, respectively. The optical band gap can be obtained by generalizing the linear part of the (*αhν*)*^r^-hν* plot to zero optical absorption. Figure 10a shows UV-Vis absorption spectra of TiO_2_ and the prepared TiO_2_/C/Ag nanocomposite, in which no difference is observed between the two spectra. According to the Tauc plot of TiO_2_ and the TiO_2_/C/Ag nanocomposite, the direct band gap was obtained as 3.13 eV and 3.20 eV for TiO_2_ and TiO_2_/C/Ag samples, respectively. Therefore, the presence of carbon and Ag particles does not have an impact on the band gap values.

### 3.2. The Ability of TiO_2_/C/Ag Nanocomposite in the Reduction of MB, CR, and 4-NP

**Figure 11:**
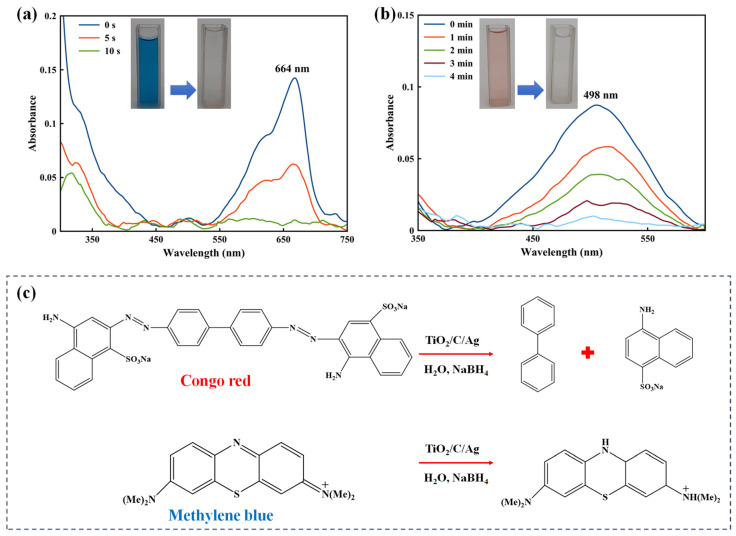
(**a**) UV-Vis spectra of (**a**) MB and (**b**) CR reduction by TiO_2_/C/Ag catalyst and NaBH_4_. Conditions: 25 mL of NaBH_4_ (5.3 × 10^−3^ M), 25 mL of MB (3.1 × 10^−5^ M), 25 mL of CR (1.44 × 10^−5^ M), and 0.008 g of TiO_2_/C/Ag. (**c**) Mechanism for the reduction/degradation of CR and MB with the TiO_2_/C/Ag nanocomposite.

Developing a facile and fast method is necessary for reducing MB and CR from water resources. For this purpose, the catalytic reduction of MB and CR was examined using a hydrogen source of NaBH_4_ and a small amount of the TiO_2_/C/Ag nanocomposite at ambient conditions. This process was investigated by detecting the reduction of UV-Vis absorption peaks at λ_max_ of ~664 nm (MB) and ~498 nm (CR). By adding fresh NaBH_4_ to the mixture of the nanocomposite and dyes, both UV absorption peaks completely disappeared after 10 s and 4 min, respectively (Figure 11a,b), suggesting a reduction/degradation of dyes. It is clear that the catalyst has better performance for MB reduction. According to the zeta potential result, MB can be adsorbed on the negative surface of TiO_2_/C/Ag due to the attraction of electrostatic interaction, improving the catalytic degradation/reduction reaction [74]. CR is a negatively charged dye that can reduce its adsorption on the TiO_2_/C/Ag nanocomposite compared with MB [75]. Figure 11c exhibits a mechanism for the catalytic reduction/degradation of MB and CR. The reduction of the dyes can occur through two steps by a TiO_2_/C/Ag nanocatalyst [9,76,77]: in the first step, dissociation of NaBH_4_ produces borohydride ions (BH_4_^−^), which can adsorb on the nanocatalyst. In the second step, the nanocatalyst accepts electrons from BH_4_^−^ and transfers them to dyes adsorbed onto the nanocatalyst through π-π stacking interactions, leading to the reduction of dyes. Eventually, the reduced dye is released from the nanocatalyst. The presence of Ag particles reduces the kinetic barrier in the dye reduction process by transferring electrons from BH_4_^−^ to MB or CR dye. In Table 2, the catalytic performance of the as-fabricated nanocomposite for reducing MB and CR dyes was compared with other nanocomposites, including Ag particles and TiO_2_ NPs. As is clear in Table 2, the TiO_2_/C/Ag nanocomposite indicates better catalytic activity, and the reduction of MB and CR with the prepared nanocomposite was performed in a shorter time.

**Figure 12:**
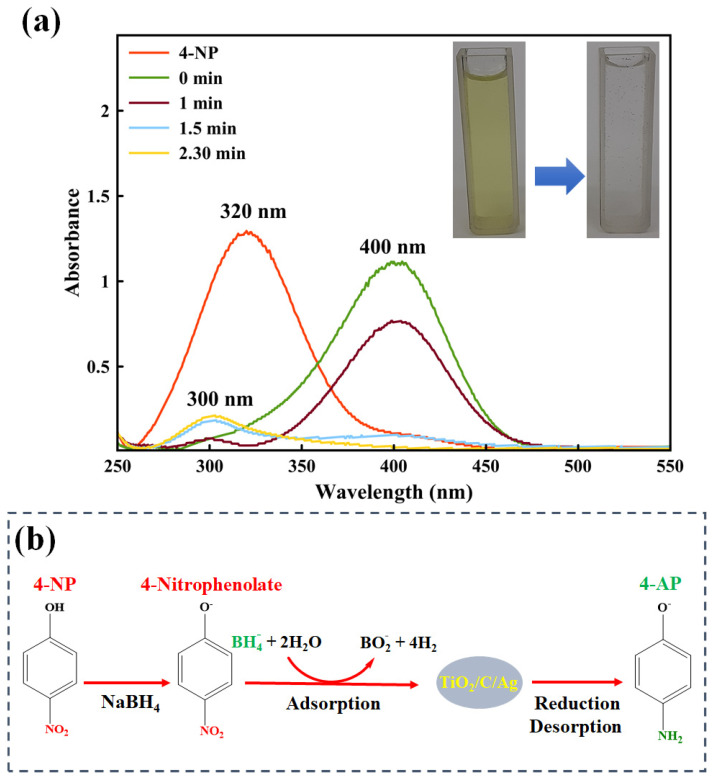
(**a**) UV-Vis spectra of 4-NP reduction. Conditions: 25 mL of NaBH_4_ (250 mM), 25 mL of 4-NP (2.5 × 10^−3^ M). (**b**) Mechanism for the 4-NP reduction using TiO_2_/C/Ag nanocomposite.

Nitroaromatic molecules are toxic organic compounds that are widely applied in laboratories and industries. These compounds can create serious hazardous problems in the human health and environment at even low concentrations. The catalytic reduction of nitrophenols to aminophenols is important, especially for pharmacology industries. Aminophenol is a fundamental precursor for preparing drugs such as acetanilide and phenacetin. Therefore, the catalytic activity of the TiO_2_/C/Ag nanocomposite was examined for the reduction of 4-NP using a NaBH_4_ reducing agent. The reduction reaction was surveyed through UV-Vis measurements, as shown in Figure 12a. According to Figure 12a, a maximum absorption peak at 320 nm is observed for aqueous 4-NP with a yellow color [86,87]. After adding a fresh NaBH_4_ solution, the light yellow color of 4-NP changed to dark yellow and the absorption peak shifted to 400 nm, suggesting 4-nitrophenolate ions formation in alkaline conditions [88,89]. After adding the TiO_2_/C/Ag nanocomposite, the absorption peak at 400 nm was reduced and a peak at 300 nm appeared, which is characteristic of 4-aminophenol (4-AP). The absorption peak at 400 nm completely disappeared after 2.30 min and the dark yellow color turned to colorless. Figure 12b displays a schematic for the 4-NP reduction reaction. When the TiO_2_/C/Ag nanocomposite is added to the reaction system, Ag particles accept the electron from BH_4_^−^ and it is transferred to the absorbed 4-NP on the nanocomposite. Finally, the produced 4-AP is desorbed from the nanocomposite surface and a free surface will be provided for another run of the catalytic reaction [9,35,77,90]. The catalytic performance of the TiO_2_/C/Ag nanocomposite for 4-NP reduction was compared with other reported catalysts, including Ag particles (Table 3).

TiO_2_ NPs are commonly used in dye removal because of their adsorption ability, increasing the removal rate [91,92,93]. In addition, the composition of carbon with TiO_2_ NPs can provide a synergetic effect on the adsorption of dyes. It can also create a porous structure with a high surface area for the adsorption of dye molecules and 4-NP [35,94,95].

The catalytic reduction/degradation of CR, MB, and 4-NP was tested in the absence of a catalyst (Table 4). It was found that the reaction between 4-NP and NaBH_4_ was not complete even after 2 h. In addition, using TiO_2_/C as the catalyst leads to a longer catalytic reduction/degradation of CR, MB, and 4-NP compared with TiO_2_/C/Ag, suggesting that the presence of Ag particles can speed up the catalytic reaction.

**Table 3:** Comparison of the catalytic performance of the TiO_2_/C/Ag nanocomposite with other reported nanocomposites, including Ag particles, for the reduction of 4-NP.

Catalyst	Concentration of 4-NP	Concentration of NaBH_4_	Reduction Time	Ref.
Ag/N-RGO	0.2 mM	0.2 M	400 s	[96]
Ag/MWCNTs–chitosan	0.10 mM	5 mM	5 min	[97]
Calcinated TiO_2_/Ag core–shell	5 mM	0.02 M	13 min	[98]
Ag/TiO_2_	15 mg/L	0.02 M	50 min	[99]
TiO_2_/C/Ag	2.5 × 10^−3^ M	250 mM	2.30 min	This work

### 3.3. Catalyst Recyclability

**Figure 13:**
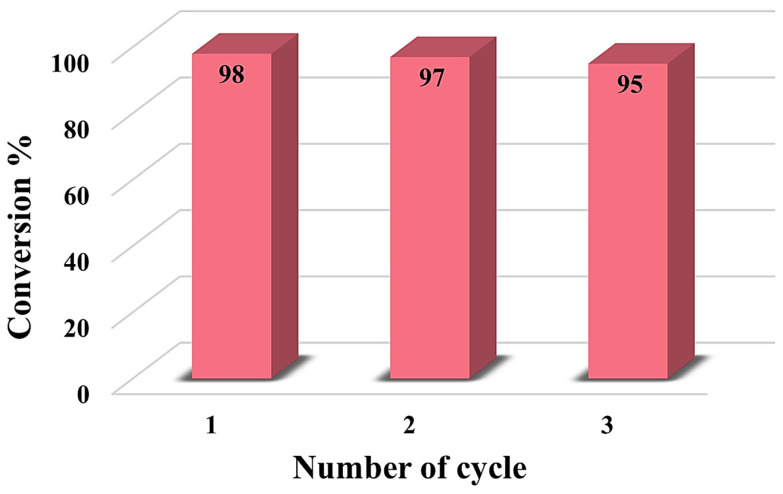
The reusability of the TiO_2_/C/Ag nanocomposite for the reduction of CR using NaBH_4_.

**Figure 14:**
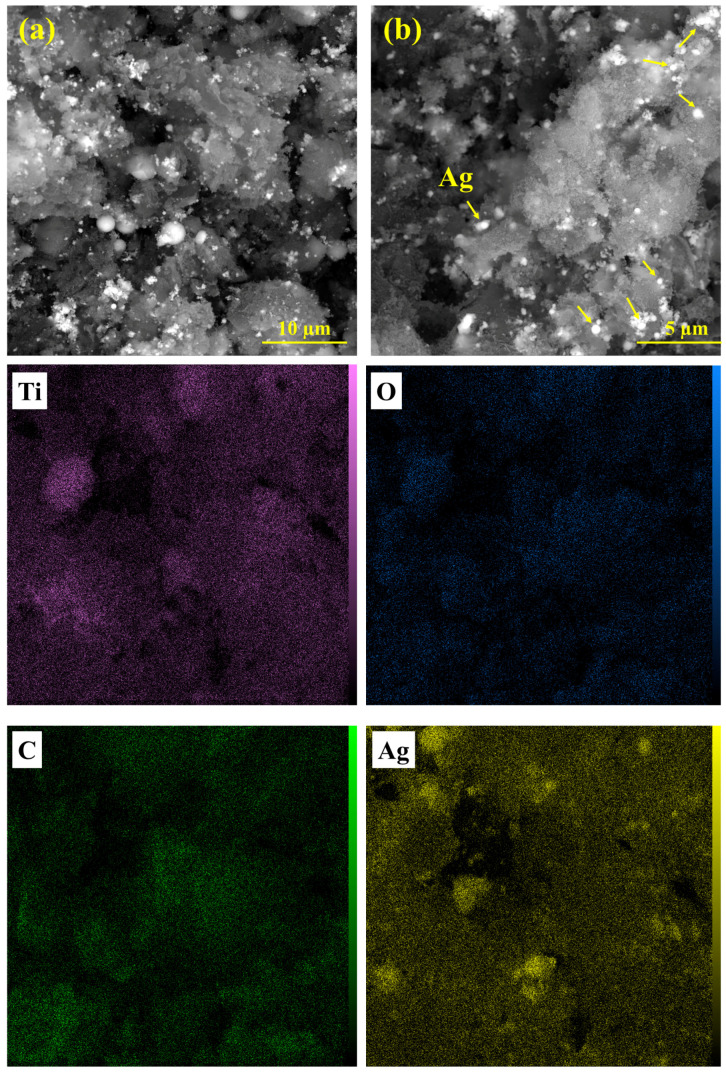
(**a**,**b**) SEM images and elemental mapping analysis of the TiO_2_/C/Ag nanocomposite after catalytic reaction.

The reusability of the TiO_2_/C/Ag nanocomposite was investigated through CR reduction. After each run, the catalyst was collected from the reaction mixture using centrifugation, washed with DI water three times, and dried at 80 °C. A slight reduction in catalyst efficiency was observed after three times (Figure 13). After the catalytic reaction, the presence of Ag particles and morphology of the nanocomposite were investigated through SEM images and elemental mapping analysis (Figure 14). The presence of Ag particles can be observed in SEM images. In addition, the Ti, C, O, and Ag elements are present even after the catalytic reaction.

## 4. Conclusions

In the present work, laser ablation in ambient air was used as a simple and fast method to prepare lignin-derived carbon and Ag particles, and then they were composited with TiO_2_ NPs (TiO_2_/C/Ag) through a stirring method. The morphology and structure of the catalyst were characterized using XRD, EDS, FT-IR, Raman, BET, and SEM analyses. The observations deduced from the Raman spectrum and FESEM micrographs substantiated the formation of a hybrid of Ag particles and TiO_2_ NPs. It was found that lignin can be successfully used as a carbon resource for preparing carbon nanostructures with significant surface areas. The composition of Ag particles and lignin-derived carbon with TiO_2_ NPs improved the surface area and average pore diameter. It can provide active sites for catalytic reactions. The catalytic efficacy of the prepared nanocomposite was examined in MB and CR reduction process. It was found that the nanocomposite can successfully reduce both dyes during 10 s (MB) and 4 min (CR). The catalytic activity of the TiO_2_/C/Ag nanocomposite was also examined for the reduction of 4-NP using a NaBH_4_ reducing agent, suggesting the complete reduction of 4-NP to 4-AP after 2.30 min. The TiO_2_/C/Ag heterogeneous catalyst is easy to recycle and the catalytic activity has not decreased significantly and has maintained its structure after three times of recycling. This study will shed light on the environmental applications of nanocatalysts and also the valorization of lignin as a natural source for the preparation of carbon. According to reports, TiO_2_-based nanocomposites are widely used for photocatalytic wastewater reactions, which occur over a long time. These nanocomposites are also used for the catalytic reduction/degradation of MB, CR, and 4-NP, which provides lower reaction times compared with photocatalytic reactions.

## Figures and Tables

**Table 1:** Surface area and porosity information of the samples.

Sample	Surface Area (m^2^/g)	Total Pore Volume (cm^3^/g)	Mean Pore Diameter (nm)
Lignin	0.77	0.0025	12.9
Carbon	25.53	0.1671	26.2
TiO_2_/C/Ag	39.49	0.4751	48.1

**Table 2:** Comparison of the catalytic performance of the TiO_2_/C/Ag nanocomposite with other reported nanocomposites, including Ag particles, for the reduction of MB and CR.

Pollutant	Catalyst	Concentration of Pollutant	Concentration of NaBH_4_	Dye Removal Time	Ref.
MB	Ag NPs/silica spheres	2 × 10^−5^ M	1 × 10^−2^ M	7.5 min	[78]
	Ag NPs	0.001 M	0.1 M	13 min	[79]
	Ag/GO	0.030 mM	500 mM	7 min	[80]
	Ag/silicon zeolite nanoparticle	0.06 mM	60 mM	4 min	[81]
	Ag/RGO	30 mg/L	0.1 M	10 min	[82]
	TiO_2_/C/Ag	3.1 × 10^−5^ M	5.3 × 10^−3^ M	10 s	This work
CR	Ag NPs@hollow mesoporous carbon spheres	0.086 mM	0.5 M	10 min	[83]
	Ag NPs	1 mM	10 mM	15 min	[84]
	AgNPs/holocellulose nanofibrils	30 mg/L	400 mg/L	30 min	[85]
	TiO_2_/C/Ag	1.44 × 10^−5^ M	5.3 × 10^−3^ M	4 min	This work

**Table 4:** The effect of the presence of the catalyst and NaBH_4_ on the catalytic reduction/degradation of MB, CR, and 4-NP.

Pollutant	Catalyst	NaBH4	Time
4-NP	-	250 mM	120 min ^a^
	TiO_2_/C (8 mg)	250 mM	120 min ^a^
	TiO_2_/C/Ag (8 mg)	250 mM	2.30 min
CR	-	5.3 mM	35 min
	TiO_2_/C (8 mg)	5.3 mM	20 min
	TiO_2_/C/Ag (8 mg)	5.3 mM	4 min
MB	-	5.3 mM	2 min
	TiO_2_/C (8 mg)	5.3 mM	1 min
	TiO_2_/C/Ag (8 mg)	5.3 mM	10 s

^a^ Not complete.

## Data Availability

The original contributions presented in the study are included in the article.

## References

[1] Mashkoor F., Nasar A. (2020). Magsorbents: Potential candidates in wastewater treatment technology-A review on the removal of methylene blue dye. J. Magn. Magn. Mater..

[2] Katheresan V., Kansedo J., Lau S.Y. (2018). Efficiency of various recent wastewater dye removal methods: A review. J. Environ. Chem. Eng..

[3] Su C.X.-H., Low L.W., Teng T.T., Wong Y.S. (2016). Combination and hybridisation of treatments in dye wastewater treatment: A review. J. Environ. Chem. Eng..

[4] Naseem K., Farooqi Z.H., Begum R., Irfan A. (2018). Removal of Congo red dye from aqueous medium by its catalytic reduction using sodium borohydride in the presence of various inorganic nano-catalysts: A review. J. Clean. Prod..

[5] Das J., Velusamy P. (2014). Catalytic reduction of methylene blue using biogenic gold nanoparticles from *Sesbania grandiflora* L.. J. Taiwan Inst. Chem. Eng..

[6] Ahmad A., Mohd-Setapar S.H., Chuong C.S., Khatoon A., Wani W.A., Kumar R., Rafatullah M. (2015). Recent advances in new generation dye removal technologies: Novel search for approaches to reprocess wastewater. RSC Adv..

[7] Adesanmi B.M., Hung Y.-T., Paul H., Huhnke C. (2022). Comparison of dye wastewater treatment methods: A review. GSC Adv. Res. Rev..

[8] Anis S.M., Hashemi S.H., Nasri A., Sajjadi M., Eslamipanah M., Jaleh B. (2022). Decorated ZrO_2_ by Au nanoparticles as a potential nanocatalyst for the reduction of organic dyes in water. Inorg. Chem. Commun..

[9] Jaleh B., Mousavi S.S., Sajjadi M., Eslamipanah M., Maryaki M.J., Orooji Y., Varma R.S. (2023). Synthesis of bentonite/Ag nanocomposite by laser ablation in air and its application in remediation. Chemosphere.

[10] Veisi H., Moradi S.B., Saljooqi A., Safarimehr P. (2019). Silver nanoparticle-decorated on tannic acid-modified magnetite nanoparticles (Fe_3_O_4_@TA/Ag) for highly active catalytic reduction of 4-nitrophenol, Rhodamine B and Methylene blue. Mater. Sci. Eng. C.

[11] Sen I.K., Maity K., Islam S.S. (2013). Green synthesis of gold nanoparticles using a glucan of an edible mushroom and study of catalytic activity. Carbohydr. Polym..

[12] Dasog M., Hou W., Scott R.W. (2011). Controlled growth and catalytic activity of gold monolayer protected clusters in presence of borohydride salts. Chem. Commun..

[13] Qi L., Zhang K., Qin W., Hu Y. (2020). Highly efficient flow-through catalytic reduction of methylene blue using silver nanoparticles functionalized cotton. Chem. Eng. J..

[14] Naushad M., Ahamad T., Khan M.R. (2022). Remediation of wastewater containing 4-nitrophenol using ionic liquid stabilized nanoparticles: Synthesis, characterizations and applications. Chemosphere.

[15] Lajevardi A., Yaraki M.T., Masjedi A., Nouri A., Sadr M.H. (2019). Green synthesis of MOF@Ag nanocomposites for catalytic reduction of methylene blue. J. Mol. Liq..

[16] Sana S.S., Haldhar R., Parameswaranpillai J., Chavali M., Kim S.-C. (2022). Silver nanoparticles-based composite for dye removal: A comprehensive review. Clean. Mater..

[17] Jayapriya M., Arulmozhi M. (2021). Beta vulgaris peel extract mediated synthesis of Ag/TiO_2_ nanocomposite: Characterization, evaluation of antibacterial and catalytic degradation of textile dyes-an electron relay effect. Inorg. Chem. Commun..

[18] Zhang L., Han M., Tan O.K., Tse M.S., Wang Y.X., Sze C.C. (2013). Facile fabrication of Ag/C-TiO_2_ nanoparticles with enhanced visible light photocatalytic activity for disinfection of Escherichia coli and Enterococcus faecalis. J. Mater. Chem. B.

[19] Ng H.M., Leo C., Abdullah A. (2017). Selective removal of dyes by molecular imprinted TiO_2_ nanoparticles in polysulfone ultrafiltration membrane. J. Environ. Chem. Eng..

[20] Rath P., Priyadarshini B., Behera S., Parhi P., Panda S., Sahoo T. (2019). Adsorptive removal of Congo Red dye from aqueous solution using TiO_2_ nanoparticles: Kinetics, thermodynamics and isothermal insights. AIP Conf. Proc..

[21] Sathiyan K., Bar-Ziv R., Mendelson O., Zidki T. (2020). Controllable synthesis of TiO_2_ nanoparticles and their photocatalytic activity in dye degradation. Mater. Res. Bull..

[22] Calvo-Flores F.G., Dobado J.A. (2010). Lignin as renewable raw material. ChemSusChem.

[23] Meng Y., Lu J., Cheng Y., Li Q., Wang H. (2019). Lignin-based hydrogels: A review of preparation, properties, and application. Int. J. Biol. Macromol..

[24] Wang H., Pu Y., Ragauskas A., Yang B. (2019). From lignin to valuable products-strategies, challenges, and prospects. Bioresour. Technol..

[25] Carrott P., Carrott M.R. (2007). Lignin-from natural adsorbent to activated carbon: A review. Bioresour. Technol..

[26] Zhang W., Yin J., Lin Z., Lin H., Lu H., Wang Y., Huang W. (2015). Facile preparation of 3D hierarchical porous carbon from lignin for the anode material in lithium ion battery with high rate performance. Electrochim. Acta.

[27] Saha D., Li Y., Bi Z., Chen J., Keum J.K., Hensley D.K., Grappe H.A., Meyer III H.M., Dai S., Paranthaman M.P. (2014). Studies on supercapacitor electrode material from activated lignin-derived mesoporous carbon. Langmuir.

[28] Seo J., Park H., Shin K., Baeck S.H., Rhym Y., Shim S.E. (2014). Lignin-derived macroporous carbon foams prepared by using poly (methyl methacrylate) particles as the template. Carbon.

[29] Shooshtari M., Salehi A. (2022). An electronic nose based on carbon nanotube-titanium dioxide hybrid nanostructures for detection and discrimination of volatile organic compounds. Sens. Actuators B Chem..

[30] Dikova T., Hashim D.P., Mintcheva N. (2024). Morphology and Structure of TiO_2_ Nanotube/Carbon Nanostructure Coatings on Titanium Surfaces for Potential Biomedical Application. Materials.

[31] Li Y., Sun S., Ma M., Ouyang Y., Yan W. (2008). Kinetic study and model of the photocatalytic degradation of rhodamine B (RhB) by a TiO_2_-coated activated carbon catalyst: Effects of initial RhB content, light intensity and TiO_2_ content in the catalyst. Chem. Eng. J..

[32] Martins A.C., Cazetta A.L., Pezoti O., Souza J.R., Zhang T., Pilau E.J., Asefa T., Almeida V.C. (2017). Sol-gel synthesis of new TiO_2_/activated carbon photocatalyst and its application for degradation of tetracycline. Ceram. Int..

[33] Pham T.N., Shi D., Sooknoi T., Resasco D.E. (2012). Aqueous-phase ketonization of acetic acid over Ru/TiO_2_/carbon catalysts. J. Catal..

[34] Bhadra B.N., Song J.Y., Uddin N., Khan N.A., Kim S., Choi C.H., Jhung S.H. (2019). Oxidative denitrogenation with TiO_2_@porous carbon catalyst for purification of fuel: Chemical aspects. Appl. Catal. B Environ..

[35] Nasrollahzadeh M., Atarod M., Jaleh B., Gandomirouzbahani M. (2016). In situ green synthesis of Ag nanoparticles on graphene oxide/TiO_2_ nanocomposite and their catalytic activity for the reduction of 4-nitrophenol, congo red and methylene blue. Ceram. Int..

[36] Zhang J., Zhao X., Kong Q., Wang X., Lou T. (2022). Preparation of chitosan/DADMAC/lignin terpolymer and its application of dye wastewater flocculation. Polym. Bull..

[37] Cui G., Wang X., Xun J., Lou T. (2017). Microwave assisted synthesis and characterization of a ternary flocculant from chitosan, acrylamide and lignin. Int. Biodeterior. Biodegrad..

[38] Bilal B., Niazi R., Nadeem S., Farid M.A., Nazir M.S., Akhter T., Javed M., Mohyuddin A., Rauf A., Ali Z. (2022). Fabrication of guided tissue regeneration membrane using lignin-mediated ZnO nanoparticles in biopolymer matrix for antimicrobial activity. Front. Chem..

[39] Yan Q., Arango R., Li J., Cai Z. (2021). Fabrication and characterization of carbon foams using 100% Kraft lignin. Mater. Des..

[40] Adi Nugroho R., Widiyanto G., Karimah Q., Venny V., Ridhwanul Mu’izzah N., Widyo Wartono M., Pramono E. (2024). Comprehensive Study of Physicochemical Properties in Poly-(Vinylidene Fluoride) Membranes Loaded with OPEFB’s Lignin/Lignosulfonate via Phase Inversion. ChemistrySelect.

[41] Chua K.Y., Azzahari A.D., Abouloula C.N., Sonsudin F., Shahabudin N., Yahya R. (2020). Cellulose-based polymer electrolyte derived from waste coconut husk: Residual lignin as a natural plasticizer. J. Polym. Res..

[42] Gupta S., Rahini R. (2017). Electrochemical synthesis and characterization of amorphous hydrogenated carbon (aC: H) using acetonitrile as electrolyte. Electrochim. Acta.

[43] Zhang H., Wang X., Li N., Xia J., Meng Q., Ding J., Lu J. (2018). Synthesis and characterization of TiO_2_/graphene oxide nanocomposites for photoreduction of heavy metal ions in reverse osmosis concentrate. RSC Adv..

[44] Arunmetha S., Dhineshbabu N., Kumar A., Jayavel R. (2021). Preparation of sulfur doped TiO_2_ nanoparticles from rutile sand and their performance testing in hybrid solar cells. J. Mater. Sci. Mater. Electron..

[45] Agarwal U.P., McSweeny J.D., Ralph S.A. (2011). FT-Raman investigation of milled-wood lignins: Softwood, hardwood, and chemically modified black spruce lignins. J. Wood Chem. Technol..

[46] Xu J., Zhou X., Chen M., Shi S., Cao Y. (2018). Preparing hierarchical porous carbon aerogels based on enzymatic hydrolysis lignin through ambient drying for supercapacitor electrodes. Microporous Mesoporous Mater..

[47] Lin Y., Zhang Q., Deng Y., Wu Q., Ye X.P., Wang S., Fang G. (2021). Fabricating graphene and nanodiamonds from lignin by femtosecond laser irradiation. ACS Omega.

[48] Xia M., Chen W., Wu J., Chen Y., Yang H., Chen X., Zhu D., Chen H. (2021). Organic salt-assisted pyrolysis for preparation of porous carbon from cellulose, hemicellulose and lignin: New insight from structure evolution. Fuel.

[49] Niu J., Shao R., Liang J., Dou M., Li Z., Huang Y., Wang F. (2017). Biomass-derived mesopore-dominant porous carbons with large specific surface area and high defect density as high performance electrode materials for Li-ion batteries and supercapacitors. Nano Energy.

[50] Yu C., Zhu S., Xing C., Pan X., Zuo X., Liu J., Chen M., Liu L., Tao G., Li Q. (2020). Fe nanoparticles and CNTs co-decorated porous carbon/graphene foam composite for excellent electromagnetic interference shielding performance. J. Alloys Compd..

[51] Liu Y., Pan L., Chen T., Xu X., Lu T., Sun Z., Chua D.H.C. (2015). Porous carbon spheres via microwave-assisted synthesis for capacitive deionization. Electrochim. Acta.

[52] Ilie A.G., Scarisoareanu M., Morjan I., Dutu E., Badiceanu M., Mihailescu I. (2017). Principal component analysis of Raman spectra for TiO_2_ nanoparticle characterization. Appl. Surf. Sci..

[53] Challagulla S., Nagarjuna R., Ganesan R., Roy S. (2017). TiO_2_ synthesized by various routes and its role on environmental remediation and alternate energy production. Nano-Struct. Nano-Objects.

[54] Brezestean I., Tosa N., Falamas A., Cuibus D., Muntean C., Bende A., Cozar B., Berghian-Grosan C., Farcău C. (2022). Silver nanoparticle films obtained by convective self-assembly for surface-enhanced Raman spectroscopy analyses of the pesticides thiabendazole and endosulfan. Front. Chem..

[55] Braun J., Holtman K., Kadla J. (2005). Lignin-based carbon fibers: Oxidative thermostabilization of kraft lignin. Carbon.

[56] Li W., Zhang Y., Das L., Wang Y., Li M., Wanninayake N., Pu Y., Kim D.Y., Cheng Y.-T., Ragauskas A.J. (2018). Linking lignin source with structural and electrochemical properties of lignin-derived carbon materials. RSC Adv..

[57] Zhang X., Dong S., Wu W., Yang J., Li J., Shi K., Liu H. (2020). Influence of Lignin units on the properties of Lignin/PAN-derived carbon fibers. J. Appl. Polym. Sci..

[58] Yu B., Chang Z., Wang C. (2016). The key pre-pyrolysis in lignin-based activated carbon preparation for high performance supercapacitors. Mater. Chem. Phys..

[59] Jadhav S., Navarro-Mendoza R., Lozano-Sotomayor P., Galindo-Esquivel I.R., Serrano O., Peralta-Hernandez J.M. (2019). Enhanced photocatalytic activity of TiO_2_ modified with GaI toward environmental application. Inorg. Chem..

[60] Martinez-Oviedo A., Kshetri Y.K., Joshi B., Lee S.W. (2021). Surface modification of blue TiO_2_ with silane coupling agent for NO_x_ abatement. Prog. Nat. Sci. Mater. Int..

[61] Si Z., Zhang X., Liu Y., Zhou H., Chen X., Yang X., Chen H., Zhan J. (2020). Revisiting the preparation of titanium dioxide: Aerosol-assisted production of photocatalyst with higher catalytic activity than P25. J. Mater. Sci..

[62] Wang T., Zhang Y.-l., Pan J.-H., Li B.-R., Wu L.-G., Jiang B.-Q. (2019). Hydrothermal reduction of commercial P25 photocatalysts to expand their visible-light response and enhance their performance for photodegrading phenol in high-salinity wastewater. Appl. Surf. Sci..

[63] Ndong L.B.B., Ibondou M.P., Miao Z., Gu X., Lu S., Qiu Z., Sui Q., Mbadinga S.M. (2014). Efficient dechlorination of chlorinated solvent pollutants under UV irradiation by using the synthesized TiO_2_ nano-sheets in aqueous phase. J. Environ. Sci..

[64] Li T., Vongehr S., Tang S., Dai Y., Huang X., Meng X. (2016). Scalable synthesis of Ag networks with optimized sub-monolayer Au-Pd nanoparticle covering for highly enhanced SERS detection and catalysis. Sci. Rep..

[65] Liu X., Zhu H., Wu J., Wang F., Wei F. (2019). The improved photocatalytic capacity derived from AgI-modified mesoporous PANI spherical shell with open pores. Rev. Chem. Intermed..

[66] Sing K.S., Williams R.T. (2004). Physisorption hysteresis loops and the characterization of nanoporous materials. Adsorpt. Sci. Technol..

[67] Hwang N., Barron A.R. (2011). BET surface area analysis of nanoparticles. The Connexions Project.

[68] Nasri A., Jaleh B., Khazalpour S., Nasrollahzadeh M., Shokouhimehr M. (2020). Facile synthesis of graphitic carbon nitride/chitosan/Au nanocomposite: A catalyst for electrochemical hydrogen evolution. Int. J. Biol. Macromol..

[69] Eslamipanah M., Jaleh B., Mohazzab B.F., Khazalpour S., Nasrollahzadeh M., Shokouhimehr M. (2020). Facile synthesis and electrochemical hydrogen storage of bentonite/TiO_2_/Au nanocomposite. Int. J. Hydrogen Energy.

[70] Kaushik A., Gola D., Raghav J., Gupta D., Kumar A., Agarwal M., Chauhan N., Srivastava S.K., Tyagi P.K. (2022). Synthesis of silver nanoparticles using egg white: Dye degradation and antimicrobial potential. Biointerface Res. Appl. Chem..

[71] Rajamanikandan R., Shanmugaraj K., Ilanchelian M. (2017). Concentration dependent catalytic activity of glutathione coated silver nanoparticles for the reduction of 4-nitrophenol and organic dyes. J. Clust. Sci..

[72] Nasri A., Jaleh B., Daneshnazar M., Varma R.S. (2023). Sensing properties of g-C_3_N_4_/Au nanocomposite for organic vapor detection. Biosensors.

[73] Nazila Z., Rasuli R. (2018). Anchored Cu_2_O nanoparticles on graphene sheets as an inorganic hole transport layer for improvement in solar cell performance. Appl. Phys. A.

[74] Zaoui F., Sebba F.Z., Liras M., Sebti H., Hachemaoui M., Mokhtar A., Beldjilali M., Bounaceur B., Boukoussa B. (2021). Ultrasonic preparation of a new composite poly (GMA)@Ru/TiO_2_@Fe_3_O_4_: Application in the catalytic reduction of organic pollutants. Mater. Chem. Phys..

[75] Bhatia P., Nath M. (2020). Green synthesis of p-NiO/n-ZnO nanocomposites: Excellent adsorbent for removal of congo red and efficient catalyst for reduction of 4-nitrophenol present in wastewater. J. Water Process Eng..

[76] Arnawtee W.H., Jaleh B., Nasrollahzadeh M., Bakhshali-Dehkordi R., Nasri A., Orooji Y. (2022). Lignin valorization: Facile synthesis, characterization and catalytic activity of multiwalled carbon nanotubes/kraft lignin/Pd nanocomposite for environmental remediation. Sep. Purif. Technol..

[77] Ashrafi G., Nasrollahzadeh M., Jaleh B., Sajjadi M., Ghafuri H. (2022). Biowaste-and nature-derived (nano) materials: Biosynthesis, stability and environmental applications. Adv. Colloid Interface Sci..

[78] Jiang Z.-J., Liu C.-Y., Sun L.-W. (2005). Catalytic properties of silver nanoparticles supported on silica spheres. J. Phys. Chem. B.

[79] Bonnia N., Kamaruddin M., Nawawi M., Ratim S., Azlina H., Ali E. (2016). Green biosynthesis of silver nanoparticles using ‘Polygonum Hydropiper’and study its catalytic degradation of methylene blue. Procedia Chem..

[80] Tran N.T., Tu T.N., Nguyen H.T., Phan D.T. (2020). One-step and surfactant-less synthesis of highly dispersed Ag nanoparticles on graphene oxide as highly effective catalyst for removal of organic dyes. Synth. Met..

[81] Liu S., Guo Y., Yi S., Yan S., Ouyang C., Deng F., Li C., Liao G., Li Q. (2023). Facile synthesis of pure silicon zeolite-confined silver nanoparticles and their catalytic activity for the reduction of 4-nitrophenol and methylene blue. Sep. Purif. Technol..

[82] Mohamed H.G., Nour A., Abd-Elhamid A., Gohr M.S., El-Gendi H., El-Sayed R.H., El-Bardan A.A., Hossain M.K., Trukhanov A.V., Abd-Elaziem W. (2024). Enhancement of Methylene Blue Catalytic Reduction by novel green synthesized metal decorated reduced graphene oxide: Sn@rGO and Ag@rGO. J. Alloys Compd..

[83] Xu P., Wu Z., Dai W., Wang Y., Zheng M., Su X., Teng Z. (2021). Synthesis of multiple Ag nanoparticles loaded hollow mesoporous carbon spheres for highly efficient and recyclable catalysis. Microporous Mesoporous Mater..

[84] Indana M.K., Gangapuram B.R., Dadigala R., Bandi R., Guttena V. (2016). A novel green synthesis and characterization of silver nanoparticles using gum tragacanth and evaluation of their potential catalytic reduction activities with methylene blue and Congo red dyes. J. Anal. Sci. Technol..

[85] Bandi R., Alle M., Park C.-W., Han S.-Y., Kwon G.-J., Kim J.-C., Lee S.-H. (2020). Rapid synchronous synthesis of Ag nanoparticles and Ag nanoparticles/holocellulose nanofibrils: Hg (II) detection and dye discoloration. Carbohydr. Polym..

[86] Nabikhan A., Rathinam S., Kandasamy K. (2018). Biogenic gold nanoparticles for reduction of 4-nitrophenol to 4-aminophenol: An eco-friendly bioremediation. IET Nanobiotechnol..

[87] Panda J., Biswal S.P., Jena H.S., Mitra A., Samantray R., Sahu R. (2022). Role of Lewis Acid Metal Centers in Metal-Organic Frameworks for Ultrafast Reduction of 4-Nitrophenol. Catalysts.

[88] Nasrollahzadeh M., Baran T., Sajjadi M., Yılmaz Baran N., Shokouhimehr M. (2020). Bentonite-supported furfural-based Schiff base palladium nanoparticles: An efficient catalyst in treatment of water/wastewater pollutants. J. Mater. Sci. Mater. Electron..

[89] Mostafa A.M., Menazea A.A. (2020). Polyvinyl Alcohol/Silver nanoparticles film prepared via pulsed laser ablation: An eco-friendly nano-catalyst for 4-nitrophenol degradation. J. Mol. Struct..

[90] Nasrollahzadeh M., Issaabadi Z., Sajadi S.M. (2018). Green synthesis of a Cu/MgO nanocomposite by Cassytha filiformis L. extract and investigation of its catalytic activity in the reduction of methylene blue, congo red and nitro compounds in aqueous media. RSC Adv..

[91] Belessi V., Romanos G., Boukos N., Lambropoulou D., Trapalis C. (2009). Removal of Reactive Red 195 from aqueous solutions by adsorption on the surface of TiO_2_ nanoparticles. J. Hazard. Mater..

[92] Kardanzadeh M., Kazeminezhad I., Mosivand S. (2018). Electro-synthesis and characterization of TiO_2_ nanoparticles and their application in removal of congo red from water without UV radiation. Ceram. Int..

[93] Follut F., Leitner N.K.V. (2007). Radiolysis of aqueous 4-nitrophenol solution with Al_2_O_3_ or TiO_2_ nanoparticles. Chemosphere.

[94] Simonetti E.A.N., de Simone Cividanes L., Campos T.M.B., de Menezes B.R.C., Brito F.S., Thim G.P. (2016). Carbon and TiO_2_ synergistic effect on methylene blue adsorption. Mater. Chem. Phys..

[95] Cai J., Hu S., Xiang J., Zhang H., Men D. (2020). The effect of graphitized carbon on the adsorption and photocatalytic degradation of methylene blue over TiO_2_/C composites. RSC Adv..

[96] Tian Y., Cao Y.-y., Pang F., Chen G.-q., Zhang X. (2014). Ag nanoparticles supported on N-doped graphene hybrids for catalytic reduction of 4-nitrophenol. RSC Adv..

[97] Alshehri S.M., Almuqati T., Almuqati N., Al-Farraj E., Alhokbany N., Ahamad T. (2016). Chitosan based polymer matrix with silver nanoparticles decorated multiwalled carbon nanotubes for catalytic reduction of 4-nitrophenol. Carbohydr. Polym..

[98] Mahajan J., Jeevanandam P. (2019). Novel thermal decomposition approach for the synthesis of TiO_2_@Ag core-shell nanocomposites and their application for catalytic reduction of 4-nitrophenol. J. Nanopart. Res..

[99] Liao X., Zheng L., He Q., Li G., Zheng L., Li H., Tian T. (2022). Fabrication of Ag/TiO_2_ membrane on Ti substrate with integral structure for catalytic reduction of 4-nitrophenol. Process Saf. Environ. Prot..

